# Frailty and Environmental Attributes in Older Adults: Insight from an Ecological Model

**DOI:** 10.1298/ptr.R0027

**Published:** 2023-11-18

**Authors:** Kazuki UEMURA, Tsukasa KAMITANI, Minoru YAMADA

**Affiliations:** ^1^Graduate School of Rehabilitation Science, Osaka Metropolitan University, Japan; ^2^Section of Education for Clinical Research, Kyoto University Hospital, Japan; ^3^Faculty of Human Sciences, University of Tsukuba, Japan

**Keywords:** Disability prevention, Physical activity, Social capital, Health literacy, Kayoi-no-ba

## Abstract

Many studies on frailty have primarily focused on individual-level risk factors such as demographics and lifestyle. While guidelines for frailty management recommend modifications to an individual’s lifestyle, their lifestyle behaviors are significantly influenced by their surroundings. Recently, the association between frailty and environmental attributes has drawn attention as a result of the increase in evidence that multiple factors affect health conditions and behaviors associated with frailty. These findings can be organized based on an ecological model involving five nested levels that influence an individual’s behaviors, namely, an intrapersonal/individual core (age, education, and attitude), an interpersonal level (persons and groups), an organizational/institutional level (organization and workplace), a community level (natural, built, and social environments), and a system/public policy level (public policies from local to national). This study reviewed possible factors associated with frailty from the onset and its progression at each level of the ecological model and their implications regarding frailty prevention. Additionally, we introduce a policy-level approach for frailty prevention in Japan—which encourages residents to engage in the local society by participating in community places or groups that are referred to as ”Kayoi-no-ba”—and aggregate its status from a government report. This perspective on community building is consistent with the concept of an ecological model. However, few studies have verified the effects of policy- or system-level approaches on disability and frailty prevention. Further studies from an ecological perspective are needed to fulfill multilevel interventions for frailty prevention.

**F**railty is characterized by a decline in physiological reserves and an increased vulnerability with regard to ageing-related stressors^1,2)^. The prevailing operational definitions of frailty are the phenotype model proposed by Fried et al.^1)^ as well as the accumulation deficit model proposed by Rockwood et al.^3)^. Based on either model, the instruments assessing frailty can identify individuals at high risk of adverse health outcomes, such as hospitalization, disability, and mortality^4–8)^. Implementing management strategies to prevent and delay the progression of frailty before significant functional deterioration occurs is crucial in both clinical practice and public health policies.

The phenotypic approach considers frailty as a transitional state from robustness to functional decline as the predisability stage, which is distinct from the actual disability^1,9)^. Frailty is recognized as a dynamic process that includes a natural progression as well as a remission, and is not a fixed irreversible pathway that leads to disability or mortality^10)^. In a systematic review and meta-analysis of studies investigating frailty progression and remission, Kojima et al. found that 14% of the individuals improved, 29% worsened, and 57% exhibited no change in their frailty status, with a mean follow-up of 3.9 years^11)^. According to the findings of Ofori-Asenso et al., during a median follow-up of three years, 23.3% of pre-frail individuals reverted to a robust state, whereas 35.2% of frail individuals reverted to either a pre-frail or robust state^12)^. Sustained improvement in the frailty status is reported to be associated with a lower risk of future falls as opposed to individuals who do not improve and remain frail^13)^, which supports the significance of interventions targeting the management of frailty to reduce its negative consequences.

Pre-frailty, as a prodromal state, has a lower influence on adverse health outcomes than frailty^14)^ but is highly prevalent in the community. The prevalence of pre-frailty is reported to be almost half in community-dwelling older adults, whereas that of frailty is approximately 10%^15–17)^. According to population attributable fraction analyses, health outcomes such as loss of independence (frailty, 12%; pre-frailty, 19%)^18)^, dementia (frailty, 8.6%; pre-frailty, 9.9%)^19)^, and mortality (frailty, 18.6%; pre-frailty, 12.6%)^20)^ are comparably attributable to frailty and pre-frailty. If the exposure is removed, a fraction of the outcomes can be prevented, which is the population attributable fraction. These findings suggest that frailty management is essential even with regard to individuals who do not experience or exhibit signs of frailty. As a result, population-based strategies for frailty prevention are required to improve population-level outcomes as an additional preventative method to high-risk strategies for treating and reversing frailty.

Most studies investigating the risk factors associated with frailty have primarily focused on the individual level (e.g., demographics and lifestyle)^21)^, as in other medical fields. While guidelines for frailty management recommend lifestyle modifications^22)^, individuals’ health behavior depends on their surroundings. The contextual factors affecting frailty progression have not been sufficiently emphasized.

## Application of an Ecological Model for Frailty Prevention

The relationship between frailty and the characteristics of physical and social environments has gained significant traction^23)^ as a result of increasing evidence exhibiting that multilevel factors affect health conditions and behaviors associated with frailty. These findings can be organized based on an ecological model involving five nested levels that affect behavior, namely, an intrapersonal/individual core (age, education, and attitude), an interpersonal level (persons and groups), an organizational/institutional level (organization and workplace), a community level (natural, built, and social environment), and a system/public policy level (public policies from local to national)^24)^. While individual-focused interventions (education and counseling) are effective, their impact may be temporary without environments and policies that are supportive^25)^. The ecological model emphasizes the importance of considering all levels of influence to develop multilevel interventions with the greatest possibility of success^26)^.

### Intrapersonal (individual) level

**Fig. 1.**
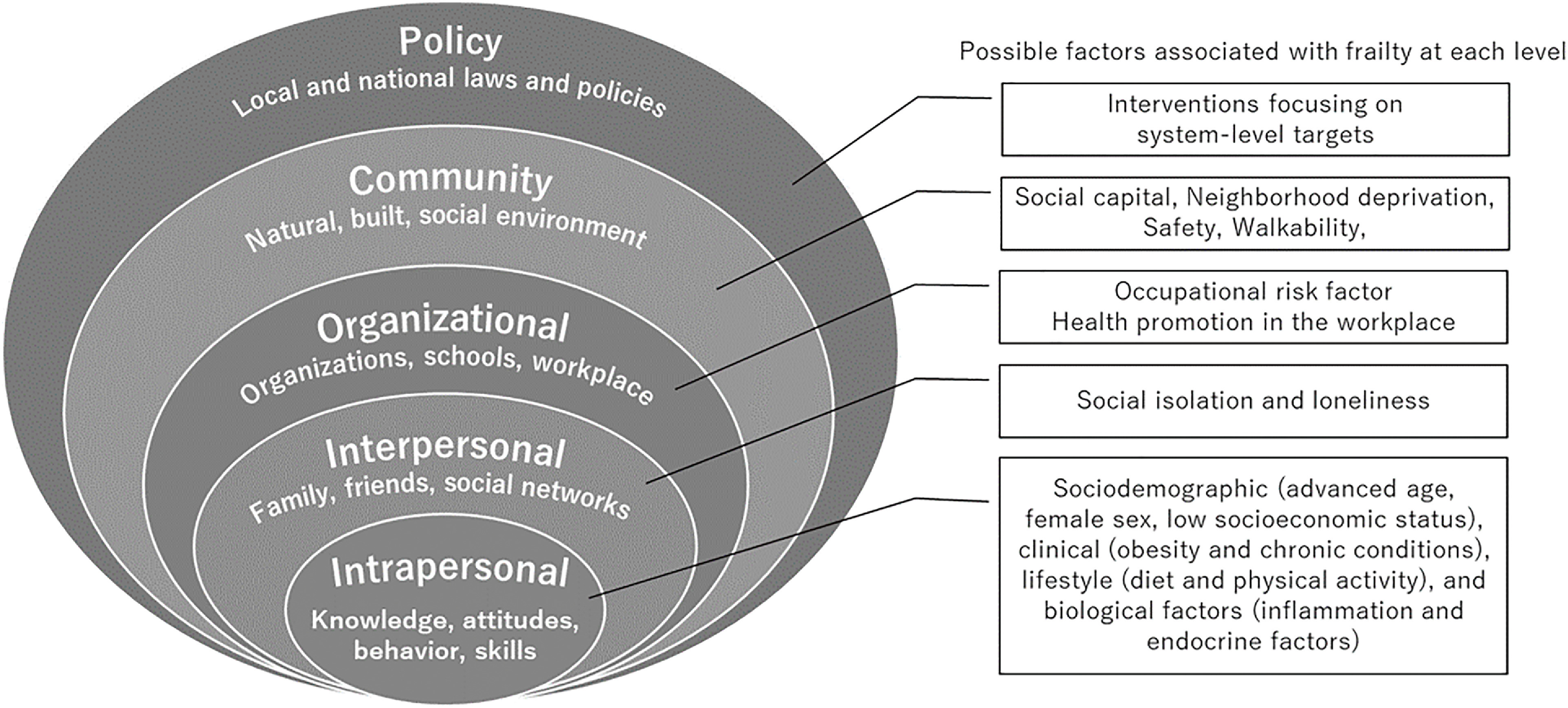
Ecological model and possible factors for frailty at each level

Figure 1 exhibits the possible factors that are associated with the onset and progression of frailty at each level of the ecological model. A wide range of risk factors for frailty onset or progression is known at the individual level, including sociodemographic (advanced age, female sex, and low socioeconomic status), clinical (obesity and chronic conditions), lifestyle (diet and physical activity), and biological (inflammation and endocrine factors)^21,27)^.

### Interpersonal level

Social factors, such as loneliness and social isolation, increase the risk of frailty onset^28)^ and progression, and decrease the likelihood of improvement to robustness^29)^. Those studies have highlighted the significance of evaluating the social networks of older adults with frailty to address the challenges they may face. Changes in health behavior (e.g., physical activity and smoking cessation) are positively associated with changes in one’s spouse’s behavior among middle-aged and older adults^30,31)^ that health behavior for frailty prevention can spread through interpersonal relationships.

### Community level

Community-level factors, such as environments that are both built and social, are associated with individuals’ health behaviors and frailty. They have drawn considerable attention and have been extensively studied for health behaviors. Several systematic reviews have reported that physical activity in older adults is positively influenced by the characteristics of the built environment, such as walkable and aesthetically pleasing neighborhoods, with access to overall and specific destinations and services^32,33)^. Recently, similar associations between built environmental attributes and frailty have been demonstrated in cross-sectional^34,35)^ and longitudinal studies^36,37)^. Walking-friendly neighborhoods protect against frailty by promoting everyday physical activity; however, the findings warrant further investigation as a result of being inconsistent^38)^.

With regard to the social environment, on a broader scale from individuals’ social networks, previous studies exhibited an inverse relationship between higher community-level civic participation as a factor of social capital and the possibility of individual frailty^39,40)^. A longitudinal study conducted over a period of 15 years reported that people living in more deprived neighborhoods were more likely to develop frailty, independent of individual demographic characteristics, health behaviors, and household wealth^41)^. One cross-sectional study reported that living in regions with intense community-based programs for frailty postponement by long-term care insurers (local governments) was associated with a lower likelihood of frailty^39)^. In these studies, a multilevel analytical approach that can differentiate the influences of community- and individual-level factors was commonly utilized to understand the contextual effects. We also reported that older adults living in communities with higher health literacy are less likely to be frail, in addition to the benefits of individual-level health literacy, in a cross-sectional study using multilevel analyses^42)^.

### Policy level

Policy-level factors were expected to have a broad influence on frailty. Some countries adopt national health policies to manage the population’s frailty at the public health and system levels^2)^. In the United Kingdom, the National Health Service (NHS) England identifies older adults with frailty by utilizing an electronic frailty index based on routine primary care coding^43)^. However, a limited number of studies have focused on policy- or system-level targets in the field of frailty, whereas most of the existing literature has focused on individual-level targets^21)^.

## Implication for Practice of Frailty Prevention from an Ecological Model

From an ecological perspective, these factors are assumed to be interconnected and to interact at multiple levels. For example, the synergistic interactions between walkability and social support in explaining physical activity among older adults suggest that the combination of peer-led walking and a walking-friendly environment might effectively increase physical activity^44)^. Additionally, the ecological framework provides a broad understanding of physical activity in multiple contexts, including occupation/work, transport, and leisure^45)^, whereas the conventional social cognitive framework (e.g., the theory of planned behavior) mainly focuses on planned activities such as sports and exercise. Frailty is also thought to be influenced in multiple contexts, including everyday activities that do not aim directly at health promotion, by a combination of individual-, interpersonal-, organizational-, community-, and policy-level factors. To address frailty, it is ideal to adopt a comprehensive strategy targeting these factors at multiple levels to promote healthy lifestyles, strengthen social support networks, improve community resources, and advocate for inclusive policies and societal attitudes toward aging. To date and as per our research, no study has investigated the effects of multilevel interventions on preventing the onset and progression of frailty. With regard to research on physical activity, the Multilevel Intervention for Physical Activity in Retirement Communities (MIPARC) study, a cluster randomized trial, demonstrated the sustained effects of a 6-month multilevel intervention among older adults living in a retirement community^46)^. It utilized knowledge and skills from the social-cognitive theory^47)^ and applied them within an ecological model, incorporating intervention components at the individual (goal setting), interpersonal (group walks), and community levels (pedestrian-advocated improvements in walkability).

The ecological model offers a practical advantage: the findings of policy- and environmental-level factors propose targets for the government to focus on when trying to enhance health outcomes^48)^, such as urban design and information on areas that need intensive intervention. In addition, this perspective fits well with translational efforts to enhance community-wide health as opposed to a small clinical focus. In Ota city, Tokyo, Japan, a community-wide intervention was implemented to prevent frailty at the population level. This intervention involved building platforms for community consultations that engaged various stakeholders and implemented multi-institutional actions. While these efforts successfully raised awareness about important terms and the project’s initiatives, they did not lead to a significant reduction in frailty at the population level after a period of two years^49)^.

## Strategies for Individual Health Professionals Using an Ecological Model

Implementing multilevel interventions can be challenging due to budgetary and practical limitations because policies and environments often lie beyond the control of individual professionals and researchers^25)^. However, the ecological model may help leverage environmental attributes to enhance the effects of interventions for individuals and small groups within the broader context of a comprehensive understanding of the factors that influence specific behaviors or outcomes. As a practical example of strategies for application by health professionals from an ecological perspective, assessing not only individual health conditions and attitudes but also social support (emotional and instrumental support) and neighborhood characteristics (e.g., walkability and access to facilities or spaces for exercise) could contribute to controlling the influence of environmental factors^50)^. Encouraging participation in community organizations and groups (not limited to sports and exercise groups) can also contribute to preventing the progression of frailty. Frailer older adults perceive their neighborhoods more negatively than those who are less frail^51)^. In such cases, it may be important to prevent negative consequences, such as further restriction of life space, by sharing information about supportive neighborhood environments with peers as well as health professionals, creating a map for taking walks, conducting an audit, and adapting environmental perceptions. Walk Score is a website that can be accessed publicly to conduct an assessment of the environment to calculate how easily accessible and walkable an address is in relation to local destinations (e.g., grocery stores and parks). This enables policymakers and healthcare professionals to assess resident walkability easily^52)^.

We previously reported using both an accelerometer and a global positioning system (GPS) sensor that the higher number of places visited, rather than the amount of time spent outside the home, was associated with individuals exhibiting greater physical activity among rural older adults^53)^. Our findings suggest that assessing and intervening in the diversity of out-of-home behavior and destinations is crucial rather than relying solely on generic instructions to walk more or spend longer periods of time outside. Built environments and their positive perceptions can influence the amount of physical activity by increasing the availability of destinations that can be visited.

Notably, the ecological approach faces criticisms and has potential weaknesses^48)^. Our current understanding of how each level of influence operates and how constructs interact across these levels is insufficient, even when behavior-specific ecological models are considered^25)^. Additionally, as individual motivation and subsequent decision-making remain prominent explanations for health behavior, it is important to acknowledge that opportunity or the environment alone is insufficient to motivate an individual’s change in behavior.

## Policy Level Approach for Frailty Prevention in Japan

In Japan, long-term care insurance policies initially focused on individual or high-risk strategies intended to prevent the progression of frailty by identifying high-risk individuals as targets for short-term and intensive interventions by health professionals. The Japanese government shifted its approach to healthcare policies for disability prevention from a high-risk strategy to a population-based strategy, by amending its Long-Term Care Insurance Law in 2014. These policies include an environmental approach that encourages residents to engage in the local society by participating in community places or groups. This perspective on community building is consistent with the concept of an ecological model.

**Fig. 2.**
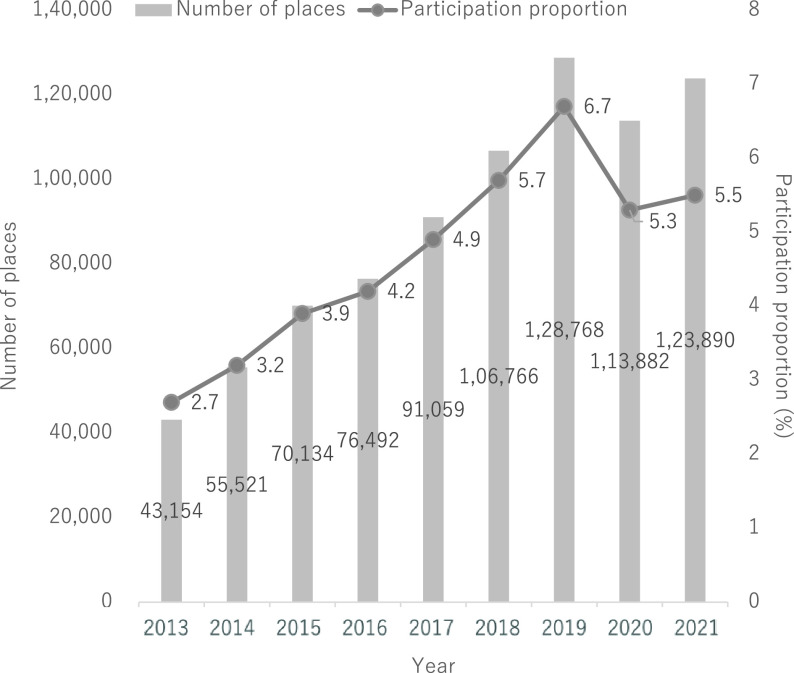
The number of places nationwide and participation proportion of “Kayoi-no-ba” from 2013 to 2021. This figure was created based on the report of the long-term care prevention and daily life support service 2021 (Ministry of Health, Labour and Welfare of Japan).

“Kayoi-no-ba” is a place where community gathering is conducted and where local older adults can participate in physical exercise, hobbies, or other activities as a voluntary group, with or without support from the local government. It is expected to contribute to health promotion and the prevention of frailty. The term “Kayoi” translates to “commuting,” and “ba” refers to “a place.” In this context, “ba” implies a central meeting point (not necessarily indicating physical location) where individuals with common interests gather naturally. “Kayoi-no-ba” has become a mainstream measure for preventing both frailty and disability. Based on a government report published in 2021^54)^, there were 123,890 places and 5.5% of older adults participated in “Kayoi-no-ba.” The most prevalent activity in “Kayoi-no-ba” was physical exercise (44.8%), followed by hobbies (16.5%) and tea parties (14.6%). The participation proportion of “Kayoi-no-ba” in the total older population had been increasing but was abruptly interrupted by the coronavirus disease outbreak in 2019 (Fig. 2).

**Fig. 3.**
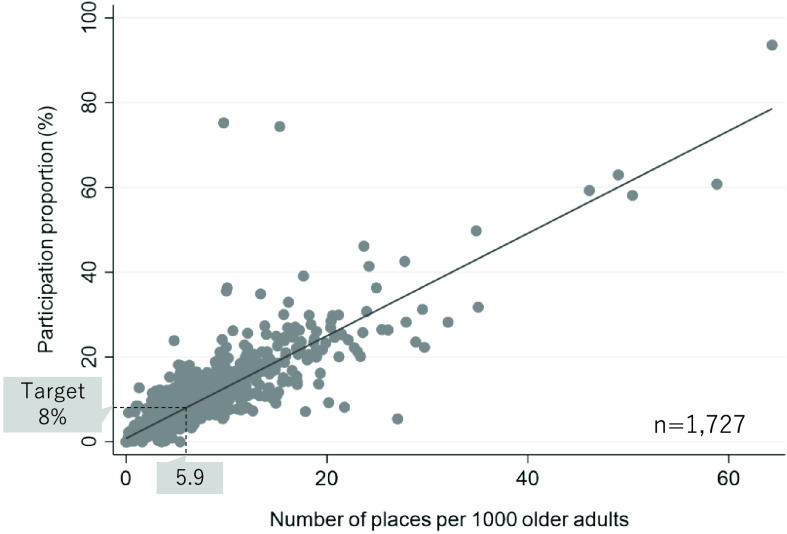
Scatterplot of the number of places and participation rate of “Kayoi-no-ba” in each local government in 2021. Areas evacuated because of the nuclear power plant accident and local governments where the participation rate was greater than 100% were excluded. This figure was created based on the report of the long-term care prevention and daily life support service 2021 (Ministry of Health, Labour and Welfare of Japan).

The Ministry of Health, Labor and Welfare reports that the status of “Kayoi-no-ba” consisted of a total of 1741 local governments (insurers) in Japan annually, which can be freely accessed online^54)^. Aggregating the information provided descriptively exhibits to us some issues and implications. For example, there was a positive correlation between the participation proportion and number of “Kayoi-no-ba” places per older adult population in each local government (Fig. 3). If the target for the participation proportion is set at 8%, as announced by the Japanese government, approximately 6 “Kayoi-no-ba” places per 1000 older adults are needed.

**Fig. 4.**
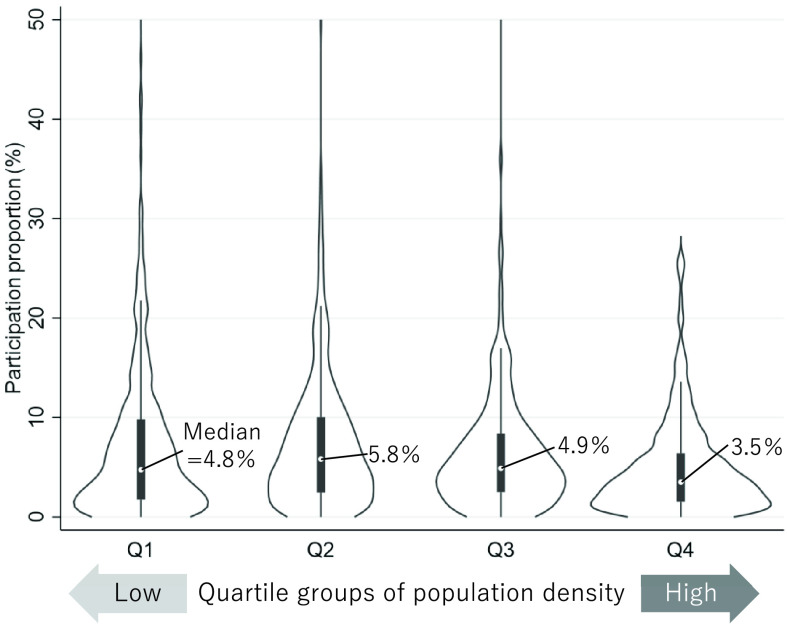
Violin plot of participation rate of “Kayoi-no-ba” in quartile groups of population density. Areas evacuated because of the nuclear power plant accident and local governments where the participation rate was greater than 100% were excluded. This figure was created based on the report of the long-term care prevention and daily life support service 2021 (Ministry of Health, Labour and Welfare of Japan) and the 2020 Japanese Census (Statistics Bureau, Ministry of Internal Affairs and Communications of Japan).

As exhibited in the violin plot in quartile groups of the population density (Fig. 4), the participation proportion of “Kayoi-no-ba” is relatively low in the local government of the group with the highest population density. Population density data were obtained from the 2020 Japanese Census (Statistics Bureau, Ministry of Internal Affairs and Communications of Japan)^55)^. Several reasons are supposed for this, such as the fact that the social network of residents is originally weaker in urban areas, and the venues for meetings (e.g., community centers) are limited and narrower when compared to the size of the population, as opposed to rural areas. It is important to identify the factors associated with a lower proportion of participation and take measures against it, especially according to local conditions, including the level of urbanization and community resources.

Some studies investigated the effects of participating in “Kayoi-no-ba” on disability occurrence at the individual level by way of an observational study utilizing propensity score matching among community-dwelling older adults^56,57)^. Observational evidence supports the potential benefits, while verification of the effects of healthcare policies of community-building events like “Kayoi-no-ba” that focus on frailty and disability prevention is limited. Therefore, academia and government must collaborate to plan healthcare policies and scientifically analyze their effectiveness. In addition, there is limited evidence of its effects on disability or frailty at the population level. Further studies are needed to explore effective policy- or system-level approaches to frailty prevention.

## Conclusion

We applied an ecological model involving five nested levels that affect behaviors to organize the risk and protective factors for frailty onset and its progression. Policy and environmental factors can be proposed as targets for the government to focus on frailty prevention, although the evidence is limited compared to individual-level factors. Meanwhile, as a practical implication, the ecological model can help health professionals leverage environmental attributes to enhance the effects of interventions, even for individuals and small groups, through a comprehensive understanding of the factors affecting frailty status. Further studies from an ecological perspective are required to develop multilevel interventions for frailty prevention.

## Conflict of Interest

All authors declare no conflict of interest.
